# Personzentrierte Wundversorgung: Legitimation und theoretisches Konzept

**DOI:** 10.1007/s00391-025-02466-w

**Published:** 2025-07-28

**Authors:** Eva-Maria Panfil, Joachim Dissemond, Julian-Dario Rembe, Bernd Assenheimer, Veronika Gerber, Christian Hafner, Peter Kurz, Martin Motzkus, Robert Strohal, Jürg Traber, Sebastian Probst

**Affiliations:** 1 Medizinische Direktion, Abteilung für Praxisentwicklung und Forschung Pflege/MTT, Universitätsspital Basel, Spitalstr. 22, 4031 Basel, Schweiz; 2 Klinik und Poliklinik für Dermatologie, Venerologie und Allergologie, Universitätsklinikum, Hufelandstraße 55, 45147 Essen, Deutschland; 3 Klinik für Gefäß- und Endovaskularchirurgie, Universitätsklinikum Düsseldorf (UKD), Heinrich-Heine-Universität (HHU), Moorenstraße 5, 40225 Düsseldorf, Deutschland; 4 Universitätsklinikum, Schule für Pflegeberufe, Otfried Müller Straße 39/1, 72076 Tübingen, Deutschland; 5 Schulung und Beratung im Wundmanagement, Anne-Frank-Str. 10, 48480 Spelle, Deutschland; 6 Venenklinik Bellevue, Brückenstr. 9, 8280 Kreuzlingen, Schweiz; 7 WPM Wund Pflege Management GmbH, Prof. Knesl Platz 11, 2222 Bad Pirawarth, Österreich; 8 Ev. Krankenhaus, Wertgasse 30, 45468 Mülheim an der Ruhr, Deutschland; 9 Abt. Dermatologie und Venerologie, LKH Feldkirch, Carinagasse 45, 6800 Feldkirch, Österreich; 10 Venenklinik Bellevue, Brückenstraße 9, 8280 Kreuzlingen, Schweiz; 11 HES-SO Fachhochschule Westschweiz, Genf, Schweiz; 12 Universitätsspital, Genf, Schweiz; 13 Universität, Genf, Schweiz; 14 Universität Monash, Melbourne, Australien; 15 Universität, Galway, Galway, Irland

**Keywords:** Wunden, Patientenzentrierte Versorgung, Patientenedukation, Patient Reported Outcome Measures, Patient Reported Experience Measures, Wounds, Patient-centered care, Patient education, Patient reported outcome measures, Patient reported experience measures

## Abstract

**Hintergrund:**

Internationale Vereinigungen fordern mit „Personzentrierter Versorgung (PZV)“ ein statt auf Krankheitsbilder auf „Menschen mit“ chronischen Krankheiten ausgerichtetes Versorgungskonzept.

**Fragestellung:**

Darstellung des Konzeptes und seiner Legitimation, bezogen auf die Versorgung von Menschen mit chronischen Wunden.

**Material und Methode:**

Narratives Review, Identifikation und Analyse von Übersichtsarbeiten sowie konzeptuellen Grundlagen, Praxisbeispiele, Diskussion einer Umsetzung.

**Ergebnisse:**

Eine PZV orientiert sich an den gesundheitlichen Bedürfnissen und Erwartungen sowie Werten und Überzeugungen der Menschen und nicht an Krankheiten, Symptomen und klinischen Daten. Inhalte sind u. a., dass die betroffenen Menschen die Unterstützung und Ausbildung erhalten, um Entscheidungen zu treffen und an ihrer eigenen Versorgung mitzuwirken. Für Evaluationen zur Umsetzung der PZV werden v. a. Teilkomponenten wie Kommunikation oder gemeinsame Entscheidungsfindung gemessen und patientenbezogene Ergebnismessungen (PROM) und Erfahrungsmessungen (PREM) genutzt. Größte Barrieren für die Umsetzung des Konzeptes sind ein auf die Akutversorgung ausgerichtetes Gesundheitssystem, mangelnder Einbezug der Patienten in Entscheidungen sowie unzureichende kommunikative Kompetenzen und krankheitsbezogene Haltungen. Eine erfolgreiche Umsetzung bedarf kultureller, struktureller und organisatorischer Maßnahmen.

**Schlussfolgerungen:**

Es braucht Überzeugung und langen Atem, um eine PZV im klinischen Alltag umzusetzen. Organisationen müssen sich darauf einlassen wollen. Die Deutsch-Österreichisch-Schweizerische Wundheilungsorganisation (WundD.A.CH) hat die Förderung der Umsetzung als einen Schwerpunkt identifiziert.

## Abstract (en)

**Background:**

International associations advocate the care model of person-centered care (PCC) that focuses on people with chronic illnesses rather than on disease patterns.

**Objective:**

To present the concept and its legitimacy concerning the care of people with chronic wounds.

**Material and method:**

A narrative review, identification and analysis of review articles as well as conceptual foundations, practical examples and discussion of implementation.

**Results:**

The PCC is based on the health needs, expectations, values and commitments of persons and not on diseases, symptoms and clinical data. Key components include providing the affected individuals with the support and education needed to make decisions and participate in their own care. Evaluations of PCC implementation primarily measure components, such as communication or shared decision making and use patient-reported outcome measures (PROM) and patient-reported experience measures (PREM). The largest barriers to implementing the concept are a healthcare system focused on acute care, a lack of patient involvement in decision making, inadequate communication skills and disease-oriented attitudes. A successful implementation requires cultural, structural and organizational measures.

**Conclusion:**

Implementing PCC into the clinical practice requires commitment and perseverance. Organizations must be willing to engage in this approach. The German-Austrian-Swiss Wound Healing Organization (WundD.A.CH) has identified the promotion of its implementation as a key priority.

In den vergangenen Jahren wurden verschiedene Versorgungskonzepte, z. B. integrierte Versorgung, Hausarztmodelle, v. a. mit dem Ziel entwickelt, Kosten im Gesundheitswesen zu reduzieren. Ein vergleichsweise neues, breit diskutiertes und präferiertes Konzept ist das der „Personzentrierten Versorgung (PZV)“. Ziel dieser Übersicht ist es, das Konzept der PZV für den Bereich „chronische Wunden“ darzustellen.

Die Versorgung von Menschen mit chronischen Wunden wie dem diabetischen Fußulkus (DFU), Ulcus cruris venosum (UCV) und Dekubitus ist von vielen Herausforderungen geprägt: steigende Inzidenzen und Prävalenzen wegen einer älter werdenden Bevölkerung, lange Wunddauer, hohe Rezidivraten, erhebliche Einschränkungen der Lebensqualität der Betroffenen und ihrer Familien sowie hohen Gesundheitskosten [19]. Weltweit wird die Prävalenz von chronischen Wunden auf etwa 2,21/1000 Menschen geschätzt [19]. Die weltweite Prävalenz von UCV beträgt 0,32 %, die Inzidenz 0,17 % (Meta-Analyse, gemessen in Personenjahre) [22]. Fast jeder zehnte Europäer leidet an Diabetes mellitus mit steigender Tendenz. Etwa 15–25 % davon entwickeln ein diabetisches Fußsyndrom (DFS). Die 5‑Jahres-Mortalität liegt bei Komplikationen des DFS vergleichbar und teilweise sogar höher als bei Krebserkrankungen [2]. Chronische Krankheiten, zu denen auch die chronischen Wunden gehören, verursachen in der Europäischen Union (EU) einen Anteil von 70–80 % der gesamten Gesundheitskosten [8].

Die Patientenversorgung wird von den Beteiligten unterschiedlich erlebt. Gesundheitsfachpersonen (GFP) (Ärzte, Pflegefachpersonen, Therapeuten etc.) erleben Patienten als „noncompliant“ und „nicht krankheitseinsichtig“ und/oder ein Versorgungssystem, welches aus engen Zeitregimen und akut ausgerichteten Kostenstrukturen besteht, multiprofessionell statt interprofessionell sowie wenig sektorenübergreifend ausgerichtet ist. Patienten fühlen sich oft unverstanden, wünschen sich mehr Zeit, bessere Kommunikation, verständliche Informationen, mehr Teilhabe und Unterstützung sowie wertschätzende Anerkennung ihrer bisher geleisteten komplexen Bewältigungsarbeit [4, 12].

## Merke.

Patienten wünschen sich wertschätzende Anerkennung ihrer bisher geleisteten komplexen Bewältigungsarbeit.

Gesundheitsfachpersonen bewerten ihre eigene Praxis durchaus als „patientenorientiert“ [16]. Im reellen Alltag wird dies jedoch unzureichend gelebt. Implizite paternalistische Haltungen beeinflussen unbewusst Kommunikation und Entscheidungen [16]. Empowerment wird als ein Hilfsprozess betrachtet, der die Patienten dazu bringt, anders zu handeln, und nicht als Umverteilung von Macht [14]. Partizipative Entscheidungsfindung (Shared Decision-Making, SDM) ist zwar rechtlich verankert, mangelnde Kompetenzen und auch Instrumente verhindern deren zuverlässige Umsetzung in der Routineversorgung [13].

## Veränderter Versorgungsbedarf

Das Gesundheitswesen im deutschsprachigen DACH-Raum ist v. a. mit dem Fokus „Akutversorgung“ organisiert. Dies führt zu Unter- und Fehlversorgung bei vielen Patienten mit chronischen Wunden, da hier andere Herausforderungen existieren [15]:

- Chronische Wunden und ihre Grunderkrankungen sind komplex und dynamisch. Betroffene und ihre Angehörigen sollten deswegen in der Lage sein, selbstständig Symptome wahrnehmen, bewerten und adäquat handeln zu können.
- Betroffene leiden lebenslang an den Grunderkrankungen, wie z. B. Diabetes mellitus oder chronische venöse Insuffizienz. Daher ist meist keine akute Umsetzung von Maßnahmen nötig, wie z. B. bei einer Gallenkolik oder einem Herzinfarkt.
- Die Therapie bedarf der erfolgreichen Behandlung und Kontrolle der Grunderkrankungen und damit in der Regel auch Lebensstiländerungen. Diese sind sehr herausfordernd, da aus alltäglichen Gewohnheiten neue – lebenslang auszuführende – Alltagshandlungen entstehen müssen, z. B. Rauchstopp oder das Tragen von Kompressionsstrümpfen.
- Alle lebenslangen Therapiemaßnahmen müssen alltagstauglich sein, d. h., in den privaten und beruflichen Alltag passen und dauerhaft umsetzbar sein. Neue Maßnahmen, wie z. B. das tägliche Inspizieren der Füße, müssen „normal“ werden und damit „automatisch“ durchgeführt werden. Es braucht Zeit, Verständnis und Unterstützung, bis diese neue Normalität erreicht ist.
- Patienten leiden nicht selten an mehr als einer chronischen Erkrankung. GFP nehmen meist nur die in ihrem Verantwortungsbereich liegende Erkrankung wahr. Patienten müssen deswegen als eigene Case Manager agieren und für sich einen Weg aus dem „Dschungel“ von verschiedenen Fachdisziplinen mit ihren Empfehlungen von Maßnahmen finden (Beispiel 1).
- Therapieziel der Patienten ist nicht immer die Wundheilung. In der Regel betreffen sie den Alltag der Betroffenen, z. B. wieder ohne Schmerzen zum Supermarkt gehen zu können, den Partner weiter zu Hause pflegen oder den Beruf ausüben zu können.

### Beispiel 1: Herausforderungen eines Menschen mit diabetischem Fußulkus.

Der als „noncompliant“ bewertete Herr Z. leidet neben dem diabetischem Fußulkus an Adipositas, Koxarthrose mit 2 künstlichen Hüftgelenken, Spinalkanalstenose und Psoriasis vulgaris. Neben der Wundambulanz sind Hausarzt, Endokrinologe, Dermatologe und Orthopäde in die Versorgung involviert. Herr Z. versucht, die Vielzahl von unterschiedlichen Therapieempfehlungen so umzusetzen, dass er als Familienvater und Ernährer seinen Rollen gerecht werden kann. Sein Arbeitgeber zeigt wenig Verständnis für die wiederholte Krankschreibung „wegen des Lochs in den Zehen“. Deswegen droht Herrn Z. der Verlust seines Arbeitsplatzes. (S. Lachappelle, Advanced Nurse Practitioner, Universitätsspital Basel)

## Personzentrierte Versorgung

Internationale Vereinigungen, z. B. WHO und European Wound Management Association (EWMA), fordern mit der Personzentrierten Versorgung (PZV) ein auf „Menschen mit“ chronischen Krankheiten ausgerichtetes Versorgungskonzept [1, 5, 25]. Der Begriff „Person“ grenzt sich dabei deutlich von den Begriffen einer Patientenorientierung, -fokussierung und -zentrierung ab. Ihnen gemeinsam sind der Fokus „Patient“ und damit die entsprechende Rolle sowie eine Konzentration auf Gesundheit und Krankheit [18].

Es gibt keine allgemeingültige Definition von PZV [11]. Die WHO [25] definiert sie als „einen Versorgungsansatz, der

- bewusst die Perspektive von Einzelpersonen, Familien und Gemeinschaften einnimmt und
- sie als Teilnehmer und Nutznießer eines vertrauenswürdigen Gesundheitssystems betrachtet, das auf ihre Bedürfnisse und Präferenzen respektvoll, mitfühlend und auf ganzheitliche Weise eingeht.

Eine PZV setzt voraus, dass die betroffenen Menschen die Ausbildung und Unterstützung erhalten, die sie brauchen, um Entscheidungen zu treffen und an ihrer eigenen Versorgung mitzuwirken. Sie orientiert sich an den gesundheitlichen Bedürfnissen und Erwartungen der Menschen und nicht an Krankheiten“.

### Merke.

Personzentrierte Versorgung orientiert sich an den gesundheitlichen Bedürfnissen/Erwartungen und nicht an Krankheiten.

Folgende Elemente sind für eine PZV zentral [9, 11]:

- Biopsychosoziale Versorgung: Eine PZV adressiert körperliche Symptome, psychische und soziale Belastungen und Beschwerden.
- Individualität: Patienten werden als „einzigartig“ mit einer individuellen Persönlichkeit, individuellen Erfahrungen und Umgang mit der Erkrankung wahrgenommen.
- Respekt: Jeder Patient hat das Recht, gehört zu werden und als autonomes Individuum und würdevoll behandelt zu werden. Verurteilende Wertungen wie „noncompliant“ oder „nicht krankheitseinsichtig“ sind obsolet, da im Fokus Erleben, Erfahrungen, Wünsche und Bedürfnisse sowie der Alltag im Rahmen der Erkrankung liegen.
- Empathie ermöglicht eine nichtwertende, aber anteilnehmende Teilhabe an den Erfahrungen und krankheitsbezogenen Leistungen im Alltag der Patienten (Beispiel 2).
- Empowerment beschreibt eine partizipative Haltung, die Patienten das Recht einräumt, mit ihren Vorstellungen an ihrer Versorgung mitzuwirken. Voraussetzung dazu sind verständliche Informationen, ausreichende Edukation und Unterstützung. (Beispiel 3).
- Shared Decision-Making bedeutet geteilte Verantwortung. GFP mit ihrer Expertise für Diagnostik und Therapie treffen gemeinsam Entscheidungen mit Patienten und ihren Angehörigen im Rahmen derer Expertise für ihre Bedürfnisse, ihren Alltag und ihren Erwartungen (Beispiel 4).
- Kommunikation, z. B. aktives Zuhören oder Motivational Interviewing [21].

### Beispiel 2: Empathie in einem Arztbrief.

Herr H. mit langjährigem Nikotinabusus (täglich eine Schachtel Zigaretten) hat erfolgreich den Rauchstopp bewältigt. Auszug aus dem Arztbrief: „Wir sind hocherfreut über den 3‑monatigen Rauchstopp und wünschen viel Energie und Kraft für die weitere Umsetzung.“ Diese Zeilen machen Herrn H. stolz und motivieren ihn für den weiteren Rauchstopp. (L. Weibel, Pflegeexperte MScN; Prof. O. Pfister; Kardiologe, Universitätsspital Basel)

### Beispiel 3: Entwickeln eines gemeinsamen Verständnisses zum Selbstmanagement.

Frau L. ist überzeugt, dass sie nichts zur Verhinderung ihres diabetischen Fußulkus (DFU) beitragen kann. Die Wundexpertin überreicht und bespricht mit ihr den Fragebogen zur Messung des Selbstmanagements „FAS-Prä-DiFuss“ (Frankfurter Aktivitätenkatalog der Selbstpflege zur Prävention des DFU). Frau L. realisiert das erste Mal ihre Möglichkeiten der Mitarbeit und kann diese nun gemeinsam mit der Wundexpertin planen. (E. Kohler – von Siebenthal, Wundexpertin, Spitex Region Interlaken AG)

### Beispiel 4: Beziehungsaufbau und Shared Decision-Making.

Herr Z. (Beispiel 1) setzt trotz mehrfacher Information die notwendige Druckentlastung nicht um. In einem langen Gespräch mit einer Wundexpertin zu Entstehung seiner Wunde, seinem Alltag, Beschwerden, Lebensqualität und persönlichen Sorgen entsteht eine Beziehung zu der Wundexpertin, in dem das „Leben mit der Wunde“ Platz findet. Herr Z. trägt als Lkw-Fahrer ganztags Arbeitsschuhe mit Schutzkappen und läuft abends barfuß, um „einfach nur frei zu sein“. Gemeinsam werden Lösungen für druckentlastende und bequeme Schuhe gesucht. Herr Z. trägt anschließend konsequent orthopädische Hausschuhe. (J. Wüthrich, Wundexpertin, Universitätsspital Basel)

Studien zeigen, dass durch eine PZV Kosten gesenkt, Symptome reduziert, Lebensqualität gesteigert und die Versorgungsqualität verbessert werden kann [10]. Für den Bereich chronische Wunden fehlen bislang entsprechende Forschungen. Problematisch ist jedoch bei allen Studien, dass es keine einheitliche Definition von PZV gibt. Tendenziell werden alle Interventionen, die die Person als solche und nicht die Krankheit adressieren, als personzentriert definiert.

## Herausforderungen für die Umsetzung einer Personzentrierten Versorgung

Weltweit versuchen Gesundheitsorganisationen und -systeme, das Konzept der PZV umzusetzen [23]. Größte Barrieren [11] sind auf Ebene der

- Patienten und Angehörigen: eine selbst gewählte oder erwartete passive Rolle sowie mangelnde Gesundheitskompetenz.
- GFP: mangelnder Einbezug der Patienten und ihrer Angehörigen beim Setzen von Behandlungszielen und Therapieentscheidungen, mangelndes Wissen zum Konzept des PZV, mangelnde kommunikative Kompetenz sowie eine zunehmend hohe Arbeitsbelastung bei immer weniger Mitarbeitenden.
- Gesundheitsorganisationen und -systeme: ein auf Akutversorgung ausgerichtetes System, fehlendes einheitliches Verständnis von PZV zwischen den Professionen, hierarchische Strukturen, Zeitmangel sowie fehlende Möglichkeiten, in der Patientenversorgung Privatsphäre herzustellen.

## Umsetzung bedarf kultureller, struktureller und organisatorischer Maßnahmen

### Merke.

Eine erfolgreiche Umsetzung geht über die Anstrengungen einzelner Personen hinaus.

Eine erfolgreiche Umsetzung bedarf kultureller, struktureller und organisatorischer Maßnahmen [5, 9]. Dazu gehören konkrete Handlungsstrategien, Voraussetzungen der Organisation sowie Evaluation der Umsetzung (Tab. 1).

**Tab. 1:** Umsetzung einer Personzentrierten Versorgung (PZV)

Handlungsstrategien	Kennen des Patienten als „Person“, z. B. Beschwerden, Krankheitserfahrungen, Wissen zur Erkrankung, Präferenzen, Alltag
	Förderung des eigenverantwortlichen Umgangs mit der Gesundheit
	Entwicklung eines gemeinsamen Verständnisses zu Erkrankung und deren Umgang, u. a. auch durch Informationsbroschüren, Videos oder Edukationsprogramme
	Schaffen von Möglichkeiten für ein gemeinsames Agieren
Voraussetzungen der Organisation	Definieren einer Vision und Schaffung von Ressourcen für deren Umsetzung
	Wahrnehmen auch der „Person“ bei den Gesundheitsfachpersonen
	Schulung und Training der Gesundheitsfachpersonen bezüglich Grundlagen von PZV, Kommunikation und Interaktion, gemeinsamer Entscheidungsfindung und kultureller Sensibilität
	Ausreichende Zeit, Flexibilität und Kontinuität
	Leitlinien, Instrumente und Dokumentationsmöglichkeiten
Evaluation	Nutzung von vorhandenen Instrumenten

Die durch jahrelange Forschung entwickelte Theorie mittlerer Reichweite zur PZV von McCormack und McCance [20] beinhaltet umfassende theoretische Überlegungen, die auch die Umsetzung des Konzeptes ermöglichen. Zentral in der Theorie ist das Verständnis, das „Person“ sowohl Patienten und Angehörige adressiert als auch GFP. Es reicht nicht, den „Menschen“ im Patienten wahrzunehmen, ohne dies auch reziprok für die GFP zu fordern. Auch hier führen Mangel an Möglichkeiten der Mitbestimmung, unzureichende Informationen und fehlende Wahrnehmung von fachlichen und persönlichen Bedürfnissen zu Frustrationen und mangelndem Engagement.

Die Theorie besteht aus 5 Ebenen:

- Makroebene: z. B. politische Rahmenbedingungen, strategische Führung und Rahmenbedingungen.
- Voraussetzungen: z. B. Klarheit über eigene Werte und Überzeugungen, berufliche Kompetenz, zwischenmenschliche Fähigkeiten.
- Kontext der Versorgung: z. B. Skill-Mix, gemeinsame Entscheidungsfindungsprozesse, Teambeziehungen, Potenzial für Innovation und Risikobereitschaft.
- Direkte personenzentrierte Prozesse: z. B. Arbeiten mit Werten und Überzeugungen von Patienten und ihren Angehörigen, Treffen gemeinsamer Entscheidungen, mitfühlend präsent sein.
- Outcomes/Ergebnisse: z. B. Einbezogensein in die Versorgung, Wohlbefinden.

Verantwortlich für eine erfolgreiche Realisierung ist damit nicht der einzelne Mitarbeitende oder eine Abteilung.

Explizite Personzentrierte Prozesse bestehen aus:

- Arbeiten mit den Werten und Überzeugungen der Patienten und ihren Angehörigen: z. B. was ist ihnen wichtig, welchen Sinn sehen sie in Geschehnissen?
- Treffen gemeinsamer Entscheidungen: Einfließen der Erfahrungen und des Wissens aller beteiligten Personen.
- Engagement im Kontakt: Akzeptanz, dass jede Situation einzigartig ist und von den Werten und Überzeugungen aller Beteiligten beeinflusst wird.
- Mitfühlend präsent sein in gezielten Aktionen und auch bei Routinetätigkeiten, verbal und nonverbal.
- Professionelle umfassende Versorgung: Beachtung aller Dimensionen einer Person, d. h. auch psychische, soziokulturelle und spirituelle.

## Evaluation

Für Evaluationen zur Umsetzung der PZV werden v. a. Teilkomponenten wie Kommunikation oder gemeinsame Entscheidungsfindung [5] gemessen sowie patientenbezogene Ergebnismessungen (Person-reported Outcome Measures, PROM) und Erfahrungsmessungen (Person-Reported Experience Measures, PREM) genutzt [3].

Die meisten Studien zur PZV konzentrieren sich auf eines der 4 folgenden Hauptthemen:

- wie Patienten oder GFP die Komponenten der PZV definieren (Definitionen),
- Art der Pflege, die Patienten wünschen, oder die Einstellungen und Werte der GFP (Präferenzen),
- Ausmaß, in dem sich die Versorgung personenzentriert anfühlt (Erfahrungen),
- Untersuchung, was als Ergebnis der PZV geschieht (Outcomes).

Englischsprachig liegt eine wachsende Zahl von validierten Instrumenten zur Messung der PZV vor [5]. Das am häufigsten verwendete Instrument ist die „Individualized Care Scale (ICS)“ [17]. Sie misst eine individualisierte Versorgung während eines Krankenhausaufenthaltes und verfügt über ausreichende psychometrische Eigenschaften auch in der deutschen Fassung.

PROM und PREM werden generisch und krankheitsspezifisch gemessen. PROM thematisieren Erfahrungen der eigenen Gesundheit und umfassen Krankheitssymptome, Behandlungsnebenwirkungen, Funktionalität und Lebensqualität [3]. Der Wound-QoL [24] misst beispielsweise die krankheitsspezifische gesundheitsbezogene Lebensqualität von Menschen mit chronischen Wunden. Es verfügt über sehr gute psychometrische Kriterien und liegt in validen Übersetzungen in über 30 Sprachen vor.

Erfahrungen mit der Gesundheitsversorgung (PREM) können durch den WOUND‑Q erfasst werden [24]. Der noch nicht in deutscher Sprache vorliegende Fragebogen erfragt u. a. die Zufriedenheit mit erhaltenen Informationen, respektvollem Umgang, interprofessioneller sowie bedürfnisorientierter Versorgung sowie Eingebundensein in die Versorgung.

## Diskussion

Eine PZV von Menschen mit chronischen Wunden ist nötig, um betroffene Personen in ihrem Sinne und in ihren Möglichkeiten zu unterstützen, ihre gesundheitlichen Bedürfnisse und Erwartungen zu erfüllen. Gleichwohl erscheint die Umsetzung für GFP, Organisationen und Gesundheitssysteme eine große Herausforderung zu sein.

### Merke.

Eigene Haltungen und Werte müssen überdacht werden.

Es gilt, Haltungen und Werte zu überdenken, sich an den Bedürfnissen betroffener Menschen zu orientieren, statt wie gewohnt an Krankheiten, Symptomen und klinischen Werten, interprofessionell zu arbeiten sowie kommunikative Kompetenzen auszubauen. Monetäre Fehlanreize, die v. a. die Versorgung mit Wundauflagen und Hilfsmitteln, aber noch zu wenig die Edukation und das Selbstmanagement der Betroffenen finanziert, erschweren die Umsetzung. Summer Meranius et al. [26] weisen zudem bei einer Umsetzung auf mögliche gegenteilige Effekte hin, z. B. erhöhte finanzielle Kosten, Ungerechtigkeiten aufgrund von Empathie sowie dem Ausschluss bestimmter Gruppen, die eine Personzentrierung überfordern würde. Bei Patienten bestehen oft noch tradierte Rollenvorstellungen, in denen paternalistische Versorgungsstrukturen erwartet und eingefordert werden [11]. Eine PZV bedeutet, diese Erwartungen zu thematisieren und gemeinsam Möglichkeiten der Entscheidungsfindung und des Selbstmanagements zu besprechen. Erste Ansätze für eine PZV sind vorhanden. Der nationale pflegerische Expertenstandard „Pflege von Menschen mit chronischen Wunden“ [7] empfiehlt, das Wissen der Patienten und ihrer Angehörigen zur Wundentstehung, ihre Erfahrungen mit der Wundbehandlung und das vorhandene Selbstmanagement zu thematisieren. Genutzt werden können verschiedene standardisierte Fragebogen, wie der FAS-Prä-DiFuss [7] und der Wound-QoL [24]. Die S3-Leitlinie der Deutschen Gesellschaft für Wundheilung und -behandlung e. V. [6] empfiehlt, die Beeinträchtigungen der Lebensqualität zu erfragen, um darauf basierend gemeinsam mit dem Patienten Therapieziele zu hierarchisieren und Behandlungspläne zu entwickeln. Ebenfalls werden Beratung und Unterstützung zu Förderung und Erhalt der Alltagskompetenzen und Lebensqualität sowie Patientenedukation thematisiert. Die EWMA [10] empfiehlt u. a. Fragen zur Schlafqualität oder Bequemlichkeit der Wundverbände zu stellen und gemeinsam mit Patienten und Angehörigen Entscheidungen für kurz- und langfristige Therapieziele und Maßnahmen zu treffen.

## Perspektive

Die Deutsch-Österreichisch-Schweizerische Wundheilungsorganisation (Wund-D.A.CH.) ist eine interprofessionelle, deutschsprachige Dachgesellschaft für alle Gruppen, welche im „Management von akuten und chronischen Wunden“ tätig sind. Wir sind von der Notwendigkeit einer PZV überzeugt und haben die Förderung der Umsetzung als ein Schwerpunktthema identifiziert. Im Projekt *POWER *(*P*ers*o*nzentrierte *W*undv*er*sorgung) werden Standards und Instrumente definiert sowie die Umsetzung in den klinischen Alltag begleitet und evaluiert.

## Fazit für die Praxis

- Chronische Krankheiten, d. h. auch Wunden, sind für Patienten und Angehörige mit anderen Herausforderungen verbunden als akute Erkrankungen.
- Internationale Organisationen empfehlen für die Behandlung das Konzept der Personzentrierten Versorgung. Zentral ist, sich an den Bedürfnissen betroffener Menschen zu orientieren, statt an Krankheiten, Symptomen und klinischen Werten.
- Merkmale sind eine empathische nichtwertende Haltung, kommunikative Kompetenzen, Arbeiten mit den Werten und Überzeugungen der Patienten und ihren Angehörigen, partizipative Entscheidungsprozesse sowie Patientenedukation.
- Die Umsetzung kann mit Instrumenten der patientenbezogenen Ergebnis- (PROM) und Erfahrungsmessungen (PREM) evaluiert werden.
- Größte Barriere für die Umsetzung ist die Organisation der Akutversorgung. Für eine erfolgreiche Umsetzung bedarf es kultureller, struktureller und organisatorischer Maßnahmen.
